# A cross-sectional network analysis of toxic leadership behaviors among nursing managers and anxiety-depression-stress among nurses: an exploratory study

**DOI:** 10.3389/fpubh.2026.1822811

**Published:** 2026-06-12

**Authors:** Xianwei Wang, Hu Jiang, Qingfang Yao

**Affiliations:** 1 Xi'an International University, Xi'an, Shaanxi, China; 2 The Third Affiliated Hospital of Zunyi Medical University (The First People's Hospital of Zunyi), Zunyi, Gui Zhou, China; 3 Chongqing Mental Health Center, Chongqing, China

**Keywords:** anxiety, depression, network analysis, nurse managers, stress, toxic leadership

## Abstract

**Background:**

Toxic leadership behavior is a significant factor contributing to depression, anxiety, and stress among nurses. However, the exact relationship and structure between these factors remain poorly understood. This study aims to investigate the association between toxic leadership behaviors and depression, anxiety, and stress.

**Objective:**

To examine the relationship between nursing managers' toxic leadership behaviors and nurses' anxiety-depression-stress emotional states, construct a network, identify core issues within the network, and explore the relationships among variables.

**Methods:**

Using convenience sampling, 1,008 nurses from Guiyang and Zunyi cities in Guizhou Province were selected as study participants between May and December 2024. Data were collected using a general information questionnaire, the Toxic Leadership Behaviors of Nursing Managers Scale, and the Depression-Anxiety-Stress Scale. Network analysis was employed to explore the correlation between nursing managers' toxic leadership behaviors and nurses' anxiety-depression-stress emotions. Network estimation was performed using the EBICglasso algorithm, centrality and bridge centrality measures were calculated, and network accuracy and stability were assessed using bootstrap methods.

**Results:**

A total of 1,008 nurses were included, with a mean age of 32.88 ± 6.03 years (range 22–59 years), of whom 94.64% were female and 78.17% held a bachelor's degree. The network structure revealed 21 significant edges between nursing managers' toxic leadership behaviors and nurses' anxiety-depression-stress emotions, of which 12 edges connected different communities and were positively correlated. The strongest bridge edge was between “humiliating behavior” and “stress,” with an edge weight of 0.27, followed by “intemperate behavior” and “depression,” with an edge weight of 0.15. Centrality analysis showed that “humiliating behavior” exhibited the highest strength, closeness, and betweenness values, making it the most influential node in the network, followed by “stress.” Bridge expected influence (BEI) analysis further identified “stress” (BEI = 0.57) and “humiliating behavior” (BEI = 0.43) as the strongest bridge nodes within their respective communities. Bootstrap analysis indicated acceptable edge weight accuracy with narrow 95% confidence intervals, and the correlation stability coefficient for BEI was 0.594, suggesting good network stability. Overall, the network model demonstrated satisfactory accuracy and stability.

**Conclusion:**

“Humiliating behavior” and “stress” were identified as core nodes and key bridge nodes within the network, representing potential targets for interventions addressing the relationship between nursing managers' toxic leadership behaviors and nurses' anxiety-depression-stress emotions. Based on these findings, hospitals may implement targeted interventions including: (1) establishing routine mental health monitoring systems for nurses with a focus on stress management; (2) developing cognitive-behavioral therapy-based stress reduction programs to enhance nurses' emotional regulation abilities. These interventions may help mitigate the negative impact of toxic leadership behaviors and safeguard nurses' mental health.

## Introduction

Nurses worldwide face significant mental health challenges, with elevated rates of anxiety, depression, and stress consistently reported across diverse clinical settings (1, 2). These psychological burdens not only compromise individual wellbeing but also contribute to adverse patient outcomes, including increased medical errors and reduced quality of care (3–5). Among the various factors influencing nurses' mental health, leadership behaviors of nursing managers have emerged as a critical determinant (6–8). Supportive leadership practices, such as transformational leadership, have been shown to alleviate team stress and foster a healthy work environment (9, 10). whereas negative leadership styles may exacerbate nurses' psychological distress and increase turnover intentions (11, 12). Therefore, identifying the specific leadership behaviors that most profoundly affect nurses' mental health is essential for developing effective interventions.

Toxic leadership behavior is a negative leadership style characterized by managers engaging in systematic, sustained, and organized destructive actions, including bullying, envy, unfair treatment, narcissism, authoritarianism, distrust of others, aggression, intimidation, and manipulation (13, 14). In recent years, research on toxic leadership behaviors among nursing managers has gradually become a focal point, primarily focusing on its negative impacts on nurses' mental health, team performance, and patient safety (15, 16). As studies (15, 17) have indicated that toxic leadership is typically characterized by authoritarianism, narcissism, abusive supervision, and unpredictability, leading to increased occupational burnout among nurses, reduced organizational commitment, and a higher risk of medical errors. Studies conducted in countries such as China and Saudi Arabia indicate that younger, highly educated nurses, as well as those working in emergency departments, are more likely to perceive toxic leadership, and that such behaviors are significantly associated with worsened conflict management (14, 18). Furthermore, toxic leadership significantly exacerbates nurses' negative emotions by undermining supportive work environments and diminishing organizational commitment. In terms of measurement tools, the ToxBH-NM scale has been translated and validated in Chinese, demonstrating good reliability and validity, making it suitable for cross-cultural research (19).

The impact of toxic leadership on nurses is profound and multidimensional, primarily affecting mental health, professional behavior, and work attitudes (20). Research indicates that nurses exposed to experiencing toxic leadership exhibit significantly increased levels of occupational burnout and emotional exhaustion (21). Notably, younger and highly educated nurses are more susceptible to perceiving these negative effects (22, 23). Toxic leadership is also associated with reduced psychological detachment, making it difficult for nurses to recover from work-related stress, which exacerbates' reality shock, particularly among newly hired nurses, who are more likely to develop turnover intentions as a result (24, 25). Furthermore, toxic leadership undermines nurses' organizational commitment, particularly affective commitment and normative commitment, while increasing avoidant conflict management behaviors, leading nurses to prefer silence or confrontation over collaborative problem-solving (26, 27). Prolonged exposure to a toxic leadership environment may also diminish nurses' moral courage and job engagement, potentially even triggering counterproductive behaviors, ultimately compromising the quality of care and patient safety (28, 29).

Negative emotions among nursing staff exhibit multidimensional characteristics, with anxiety, depression, and stress constituting the core dimensions, which collectively exert a cascading impact on nursing quality and patient safety (30). Studies indicate that nurses' anxiety primarily stems from high-intensity workloads, occupational risks, and nurse-patient conflicts, leading to increased distractibility and higher rates of operational errors (31, 32). Depression, on the other hand, is associated with low professional identity and insufficient social support (33). And prolonged depressive symptoms can reduce work engagement and increase turnover intention (34–36). Chronic stress not only exacerbates emotional exhaustion but may also impair cognitive function, thereby affecting clinical decision-making and ultimately threatening patient safety (37). The Depression, Anxiety, and Stress Scale (DASS-21) conceptualizes negative emotional states as three distinct yet interrelated dimensions, a framework that has been widely validated in nursing populations (38). Although the relationship between each dimension and leadership behaviors has been explored separately, no studies have yet investigated, there remains a lack of simultaneous investigation into how toxic leadership behaviors are differentially associated with each emotional dimension.

To address these research gaps, we require a methodological approach capable of modeling the complex, multidimensional associations between toxic leadership behaviors and emotional symptoms. Traditional variable-centered methods, which assume that relationships are uniform across samples, are ill-suited to capture the nuanced patterns of association between specific leadership behaviors and distinct emotional symptoms. Network analysis offers an alternative framework grounded in complex systems theory (39). In a network model, variables are represented as nodes, and the conditional dependencies between them as edges, allowing researchers to visualize the overall structure of associations, identify nodes that play central roles within the network, and detect bridge nodes that connect distinct clusters of variables. This approach is particularly well-suited to our research questions, as it enables us to: (1) identify which specific toxic leadership behaviors are most strongly associated with which emotional symptoms; (2) determine the relative importance of each behavior and symptom within the overall system; and (3) pinpoint potential intervention targets that may disrupt the negative cycle between toxic leadership and nurses' mental health.

Therefore, this study aims to address these gaps by employing network analysis to examine the associations between nursing managers' toxic leadership behaviors and nurses' anxiety, depression, and stress. Specifically, this study seeks to: (1) identify the structural relationships among the four dimensions of toxic leadership behaviors (intemperate, narcissistic, self-promoting, and humiliating behaviors) and the three dimensions of negative emotional symptoms (depression, anxiety, and stress); (2) determine the centrality of each node within the network to identify the most influential factors; (3) detect bridge nodes that connect the toxic leadership and emotional symptom communities, thereby revealing potential intervention targets. The findings are expected to provide evidence-based guidance for healthcare institutions to optimize nursing management practices, reduce toxic leadership behaviors, and support nurses' psychological wellbeing, ultimately contributing to improved patient safety and quality of care.

## Methods

### Design

This study is a cross-sectional analysis using an online survey and convenience sampling. The study design and reporting follow the guidelines of the Strengthening the Reporting of Observational Studies in Epidemiology (STROBE) guidelines (40).

### Sample size estimation

According to network analysis sample size estimation requirements (41), when using network analysis methods, the sample size should be at least greater than the total number of parameters (number of nodes + (number of nodes × (number of nodes – 1) / 2)). In this study, ToxBH-NM has four nodes, and DASS has three nodes, totaling seven nodes. Therefore, the minimum required sample size is 28. To ensure network stability, each parameter requires five study subjects. According to the sample size calculation formula, the minimum required sample size is 140 cases.

### Participants

From May to December 2024, nurses working in public hospitals in Guiyang and Zunyi, Guizhou Province, were invited to participate in this survey using a convenience sampling method. Inclusion criteria: (1) age ≥18 years; (2) at least 1 year of clinical nursing experience (to ensure stable perception of leadership behaviors and workplace dynamics); (3) full-time registered nurse; (4) voluntary participation with signed informed consent. Exclusion criteria: nurses undergoing further training, internship, or those on extended leave during the study period.

### Measurements

#### Demographic and sociological information

The general information questionnaire was designed by the researchers and included basic demographic information such as age (years), gender, education, professional title and etc.

#### Toxic leadership behaviors of nurse managers scale (ToxBH-NM)

The questionnaire was developed by Labrague et al. (42) as a measurement tool specific to toxic leadership behavior in nursing managers and has been translated into multiple languages. This study utilized the Chinese version translated by Yao et al. (19). The scale comprises four dimensions: impulsive behavior, narcissistic behavior, self-promoting behavior, and humiliating behavior, totaling 30 items. The Cronbach's alpha coefficient for the Chinese version is 0.951, the split-half reliability coefficient is 0.831, and the test-retest reliability coefficient is 0.959, scale-level content validity index (S-CVI) is 0.946, with item-level content validity indices (I-CVI) ranging from 0.875 to 1.000, the criterion-related validity coefficient is 0.669. In the current study sample (*n* = 1,008), the Cronbach's alpha coefficient for the total scale is 0.988, indicating good internal consistency reliability.

#### Depression anxiety and stress scale (DASS)

The questionnaire was developed by Lovibond and Lovibond (43) as a self-report measure for assessing negative emotions such as depression, anxiety, and stress, and has been translated into multiple languages. The version used in this study is the Chinese abbreviated version DASS-21 (44), which was validated for reliability and validity by Lie in a nursing population. The scale consists of three subscales: depression, anxiety, and stress, with a total of 21 items. The Cronbach's α coefficient is 0.943, and the split-half reliability coefficient is 0.915, the criterion-related validity coefficient is 0.557. In the current study sample, the Cronbach's α coefficient is 0.988.

### Data collection

Wenjuanxing (www.wjx.cn) was used to develop a web-based questionnaire, which was rigorously reviewed by two researchers. We also created a poster displaying links and QR codes, clearly indicating the populations included and excluded in this study. We distributed the questionnaire in Guiyang and Zunyi. Researchers contacted general nurses to distribute the questionnaire, sent the poster and information letter via WeChat, and the survey could be completed by clicking on the link or scanning the QR code. Completed questionnaires could be returned directly to the webpage. Backend settings cannot be submitted more than once. After data collection was completed, two researchers manually reviewed the data to exclude invalid questionnaires. Criteria for invalid questionnaires: (1) obvious logical inconsistencies in responses; (2) excessive consistency in responses (selecting the same option for 10 or more consecutive questions); (3) response time is too short (< 2 min).

### Statistical analyses

This study aims to explore the macro-level relationship between toxic leadership behaviors (four dimensions) and negative emotional symptoms (three dimensions) at the construct level. Under these circumstances, if items are phrased similarly, item-level networks are prone to local dependencies due to methodological effects; that is, high correlations between items may stem from measurement error rather than genuine construct relationships, thereby introducing spurious edges. Therefore, this study adopts dimensions as the unit of analysis to smooth out non-substantive variation and enhance the robustness and interpretability of the network structure.

Based on the above considerations, this study employs network analysis methods to achieve its research objectives. The focus is on identifying core nodes and bridging paths that may represent potential associative pathways for future hypothesis generation, rather than confirmed intervention targets. Network analysis treats variables as nodes, with conditional dependencies between them represented as edges. It can intuitively present the overall structure and local centrality characteristics of a multivariate system, a feature that aligns closely with the objectives of this study. Furthermore, network analysis does not rely on predefined binary data structures, enabling it to effectively process the nurse-level data collected in this study and making it suitable for exploring complex associations among multidimensional variables. In summary, network analysis was determined to be the most appropriate methodological approach for addressing the research questions of this study.

Descriptive statistical analysis was performed using IBM SPSS 26.0 software, and network analysis was conducted using R language (R Core Team and R Foundation Global collaboration, New Zealand) (version 4.2.1). This included network construction, centrality measurements (strength, closeness, and betweenness), centrality stability estimation. Count data were expressed as frequencies and percentages. Continuous data that followed a normal distribution or were approximately normally distributed were presented as (Mean ± SD), otherwise, the “M [P25, P75]” was used.

Network estimation: the network was constructed using the graphical minimum absolute contraction and selection operator and the extended Bayesian information criterion algorithm (EBICglasso model) from the “qgraph1.9.8” package in the R language (45, 46). In the network model, each dimension of ToxBH-NM and DASS is treated as a node, and the relationship between two nodes is referred to as an edge (41, 47). Green solid lines indicate a positive correlation between two nodes, while red solid lines indicate a negative correlation, the thickness of the edge indicates the strength of the correlation, with thicker edges indicating stronger correlations (48).

Centrality and predictability measures: these include strength (Str), closeness (Clo), betweenness (Bet), expected influence (EI), and bridge expected influence (BEI) (49). In network analysis, when both positive and negative correlations exist, strength, closeness, and betweenness are insensitive (50). In such cases, EI should be chosen to represent the role of a node in the network (51, 52). A higher EI indicates that the node is more closely connected to other nodes in the network and plays a more important role (53, 54). Predictability refers to the extent to which all other nodes in the network can predict a given node, and it is calculated using the mgm function package.

Centrality stability estimation: first, the stability of edges is assessed using the bootnet function, and the stability of the model is evaluated through 1,000 resampling iterations (41, 55). Second, we test whether the sequence of centrality metrics remains unchanged as the sample size or number of nodes in the network decreases (56). The quantitative metric is the correlation stability coefficient (CS), where CS = 0.7 represents the maximum acceptable reduction in sample size. The minimum CS should not be less than 0.25, and values above 0.5 are generally acceptable (55).

To enhance the network layout, we used the Fruchterman-Reingold algorithm in the R package “qgraph” [(Version 1.9.8/ (Epskamp, 2023)]; “To assess the robustness of the results and improve the robustness of the findings, we performed the R ‘bootnet' package [(Version 1.6/ (Epskamp, 2024)].”

## Results

### Demographic characteristics

A total of 1,059 questionnaires were initially collected. After excluding 51 invalid questionnaires (including 23 cases with excessive consistency in the responses, 18 cases with logical inconsistencies, and 10 cases where the response time was too short), 1,008 valid questionnaires were retained for final analysis, meeting the sample size requirements. The sample characteristics align closely with those of the target population.

The ages of the 1,008 nurses ranged from 22 to 59 years old (mean age: 32.88 ± 6.032 years), there were 54 male nurses and 954 female nurses, six had an associate degree, 788 had a bachelor's degree, and 214 had a master's degree or higher, 603 held junior-level titles, 355 held intermediate-level titles, and 50 held senior-level titles, 367 were on the regular staff roster, and 641 were non-regular staff (Table 1).

**Table 1:** Demographic characteristics of the participants (*n* = 1,008).

Project	*n* (%)/Mean ±SD
Age (years)	32.88 ± 6.032
Gender	
Male	54 (5.36%)
Female	954 (94.64%)
Education	
Associate degree	6 (0.60%)
Bachelor's degree	788 (78.17%)
Master's degree~	214 (21.23%)
Professional title	
Junior-level	603 (59.82%)
Intermediate-level	355 (35.22%)
Senior-level	50 (4.96%)
Employment types	
Non-permanent staff	641 (63.59%)
Permanent employee	367 (36.41%)
Nursing experience (years)	
< 5	196 (19.44%)
5–10	418 (41.47%)
10–15	246 (24.40%)
15–20	72 (7.14%)
20~	76 (7.54%)
Hospital types	
Level I hospital	14 (1.39%)
Level II hospital	482 (47.82%)
Level III hospital	512 (50.79%)
Department	
Internal medicine system	337 (33.43%)
Surgical system	238 (23.61%)
Gynecology and obstetrics system	124 (12.30%)
Pediatric system	72 (7.14%)
Outpatient and emergency system	113 (11.21%)
Critical care system	66 (6.55%)
Nursing department	16 (1.59%)
Function department	42 (4.17%)

### ToxBH-NM and DASS dimension abbreviations and scores

The assessment includes the results from both the ToxBH-NM and DASS scales, encompassing the standard abbreviations for each dimension and the participant's scores on each (Table 2).

**Table 2:** ToxBH-NM and DASS dimension abbreviations and scores.

Scale	Dimension	*M* [P25, P75]
ToxBH-NM	IB (Intemperate behavior)	16 [16, 27]
	NB (Narcissistic behavior)	9 [9, 18]
	SPB (Self-promoting behavior)	3 [3, 6]
	HB (Humiliating behavior)	3 [3, 6]
DASS	DEP (Depression)	0 [0, 4]
	ANX (Anxiety)	0 [0, 5.75]
	STR (Stress)	0 [0, 3]

### The network

**Figure 1:**
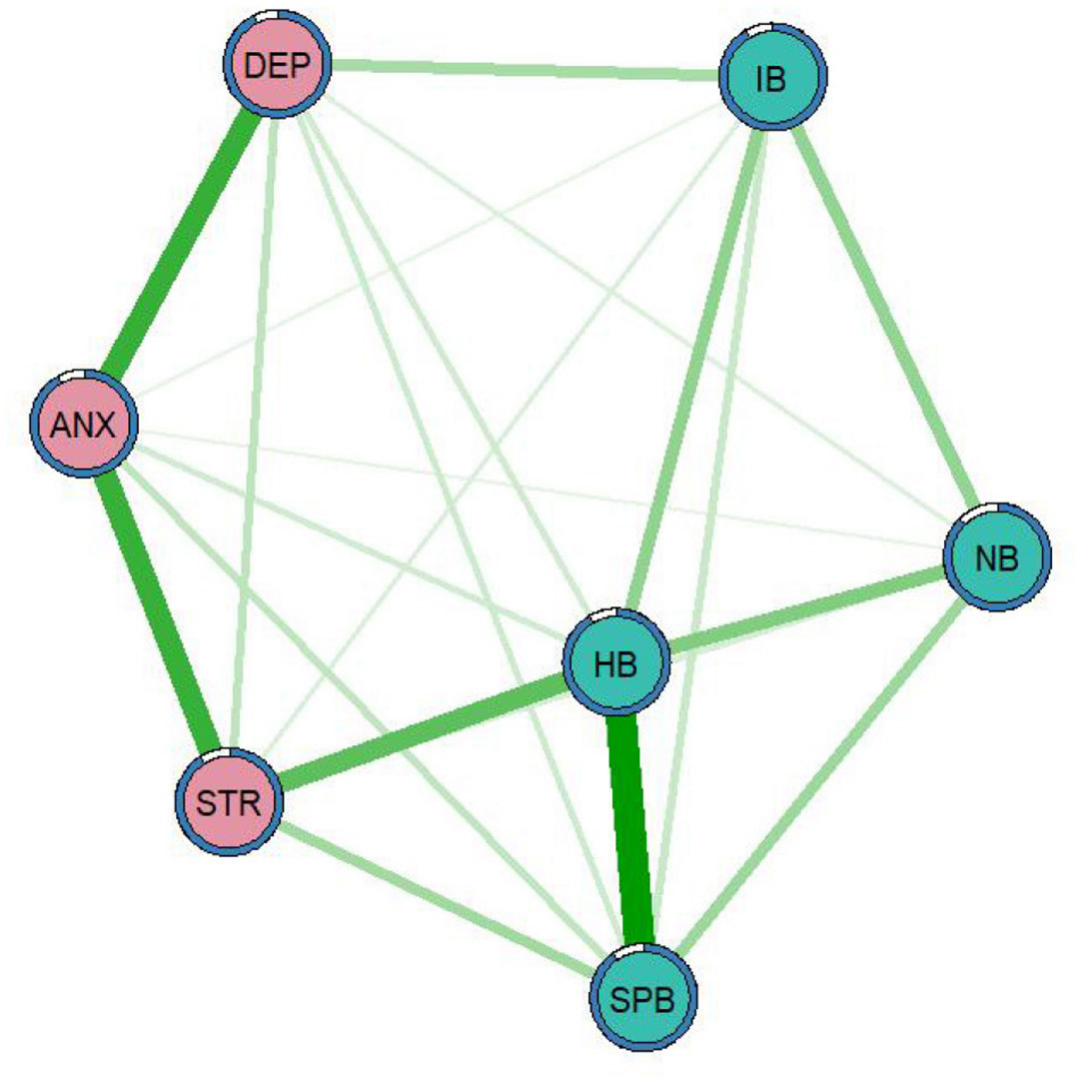
Toxic leadership behavior—depression, anxiety, and stress network model. ToxBH-NM: IB, intemperate behavior; NB, narcissistic behavior; SPB, self-promoting behavior; HB, humiliating behavior. DASS: ANX, anxiety, DEP, depression; STR, stress.

Figure 1 presents a network model of the relationship between toxic leadership behaviors experienced by clinical nurses and depression, anxiety, and stress. The network comprises a total of 21 edges with non-zero weights, 12 of which connect different communities and are all positively correlated, indicating that an increase in toxic leadership behaviors is associated with a concurrent rise in nurses' negative emotions. The strongest bridging edge is between “Humiliating Behavior” (HB) and “Stress” (STR; edge weight = 0.27), suggesting that humiliating behavior is most directly associated with nurses' stress responses; the second strongest is between “Impulsive Behavior” (IB) and “Depression” (DEP; edge weight = 0.15), indicating that impulsive leadership behavior is more likely to induce depressive emotions. These results reveal distinct association patterns between different types of toxic leadership behaviors and specific negative emotions. The specific weight values for each edge are detailed in Table 3.

**Table 3:** Network edge weights.

Variables	IB	NB	SPB	HB	DEP	ANX	STR
IB							
NB	0.18						
SPB	0.09	0.17					
HB	0.19	0.21	0.44				
DEP	0.15	0.06	0.09	0.08			
ANX	0.04	0.04	0.11	0.08	0.34		
STR	0.06	0.07	0.16	0.27	0.11	0.34	

### Accuracy and stability analysis

**Figure 2:**
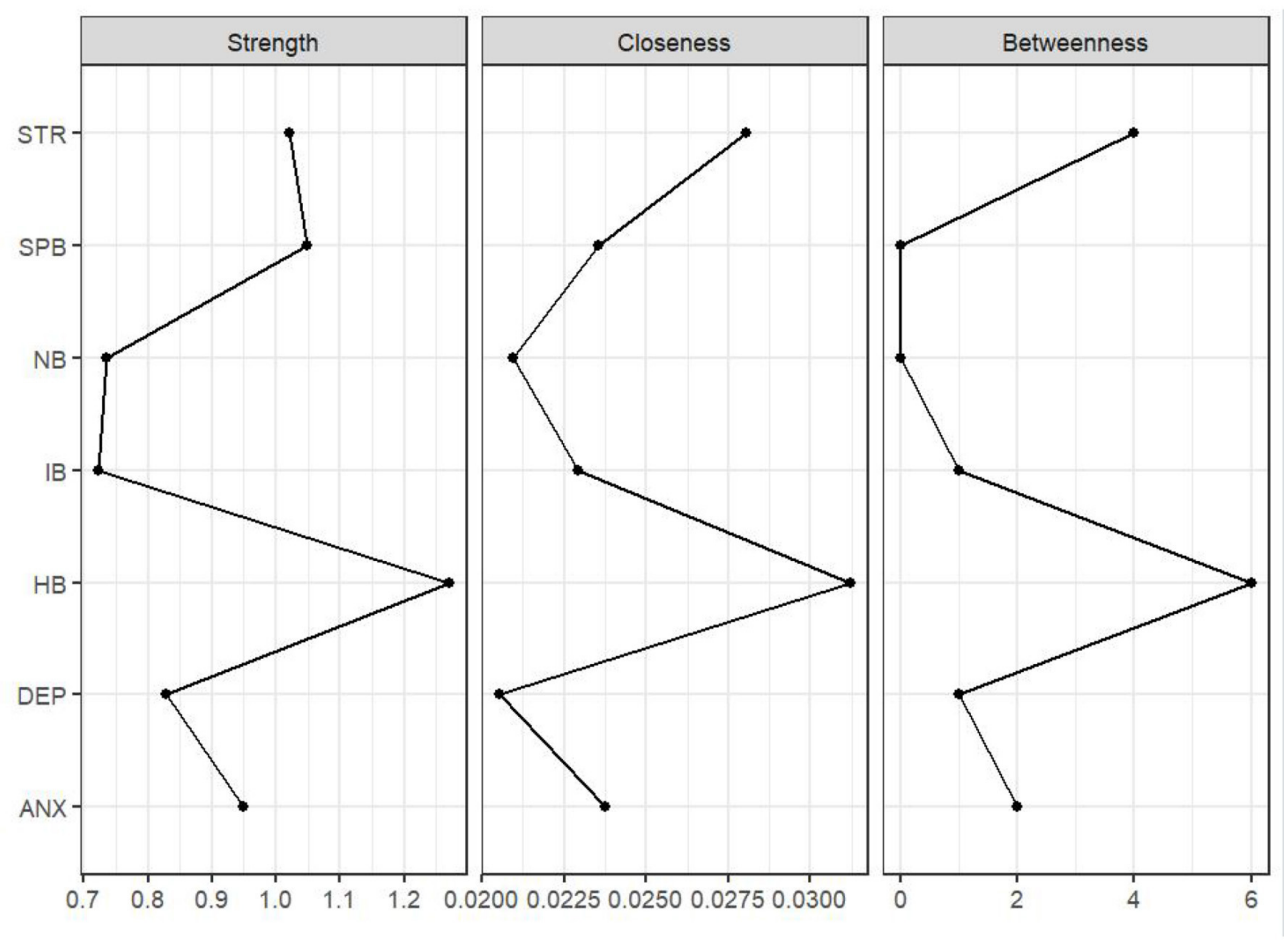
Comparison of network centrality indicators for different metrics.

Figure 2 illustrates the centrality metrics, closeness (Clo), betweenness (Bet), and strength (Str), for each node in the network model. Node HB (humiliating behavior) exhibits high levels across all three metrics, indicating that it is the most influential core node in the network, this node is not only close to other nodes (high closeness), plays a key mediating role in information transmission within the network (high betweenness), and has strong connections with other nodes (high connection strength). Node STR (stress) ranks second, also demonstrating strong network influence. In contrast, nodes NB (narcissistic behavior), IB (Intemperate behavior), and DEP (depression) have the lowest connection strength values, suggesting that these nodes have relatively limited influence within the network and occupy peripheral positions. Overall, HB is the most critical node in the toxic leadership behavior network.

**Figure 3:**
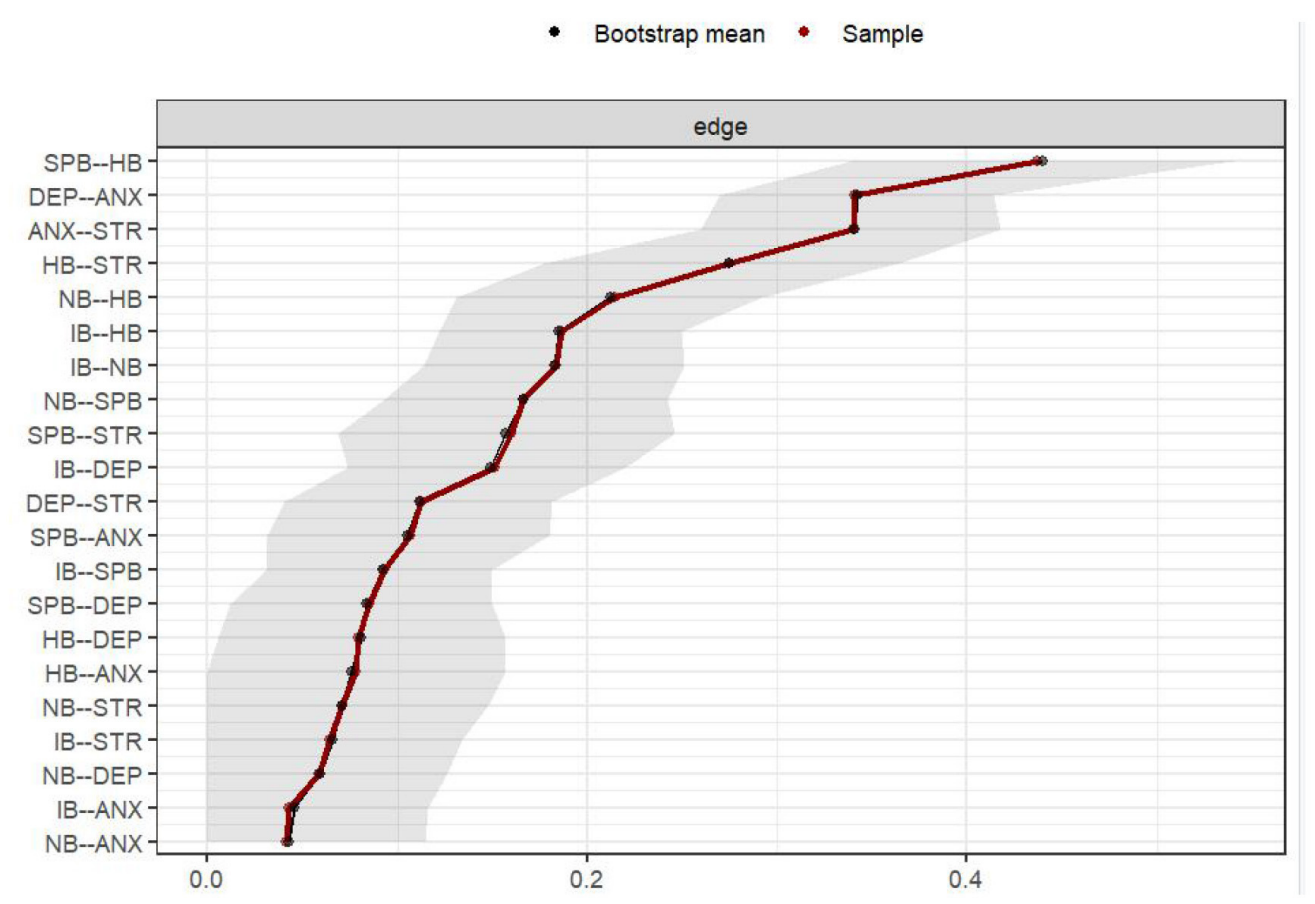
Network accuracy estimation.

Figure 3 presents the results of the stability test for network edge weights. The 95% confidence intervals calculated using the bootstrap method are relatively narrow, indicating that the estimates of edge weights are highly accurate and that the network structure exhibits good stability. This suggests that the deviation between the edge weights estimated from the current sample and the true population values is small, and the reliability and reproducibility of the research findings are statistically supported.

### Bridge centrality analysis

**Figure 4:**
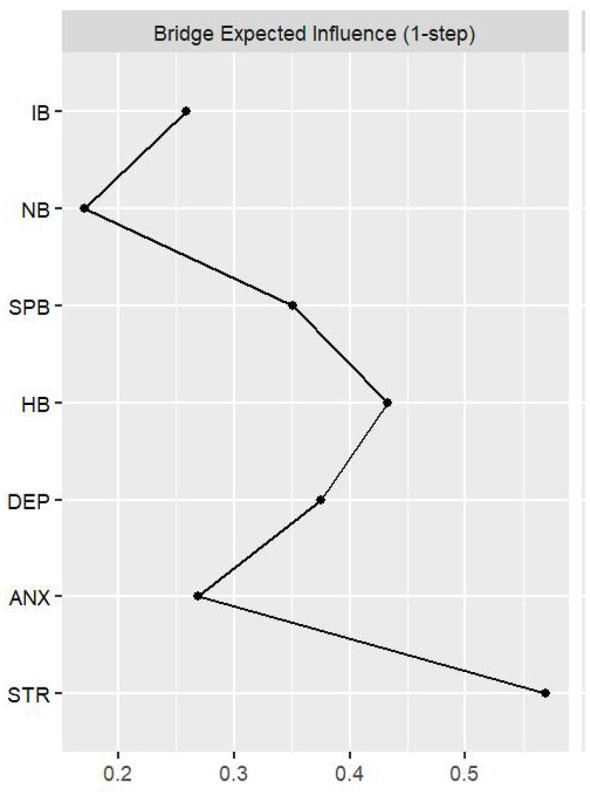
Bridge centrality analysis.

Figure 4 shows the expected influence (BEI) values of each node within its respective community, which are used to assess the importance of a node within its community. In the toxic leadership behavior community, HB “humiliating behavior” (BEI = 0.43) has the highest BEI value, indicating that it is the most influential core node in this community; In the negative emotions community, the STR “stress” (BEI = 0.57) also occupies a dominant position, suggesting that stress is the most critical emotional response node among depression, anxiety, and stress. Furthermore, the coefficient of stability (CS coefficient) for node BEI is 0.594 (≥0.25) (51, 52), indicating that the community structure of the network and the ranking of node importance are stable, and the research results are highly reliable.

### Bridge centrality stability analysis

**Figure 5:**
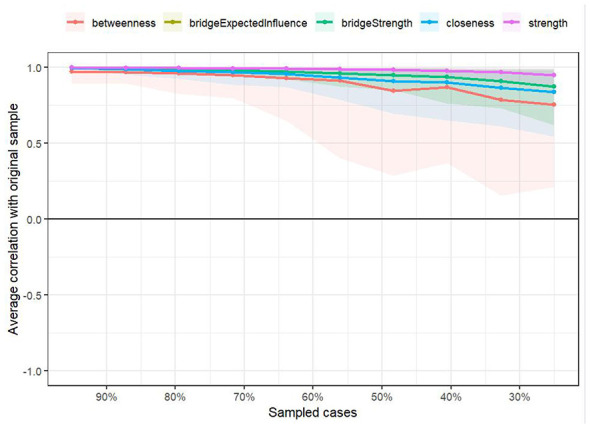
Average correlation with original sample for different centrality measures across.

Figure 5 presents the results of the stability test for network centrality metrics. The results show that bridging strength, closeness, and strength exhibit high accuracy (the lower limit of the 95% confidence interval for their estimates is ≥0.25, indicating a small deviation from the true population values), while betweenness centrality exhibits slightly lower accuracy (≥0.25, which is within an acceptable range) (41, 51). Combined with the aforementioned stability test results, it can be concluded that the network model constructed in this study possesses high accuracy and good stability.

## Discussion

The study employed network analysis to systematically examine the structural relationships between nursing managers' toxic leadership behaviors and nurses' anxiety, depression, and stress. Unlike previous research that predominantly treated toxic leadership as a unidimensional construct or examined its effects using variable-centered approaches (57, 58), our findings reveal distinct pathways: humiliating behavior emerged as the most influential dimension specifically linked to stress, while intemperate behavior showed a unique association with depression. Furthermore, stress served as the critical bridge connecting leadership behaviors to emotional symptoms. These findings challenge the assumption that toxic leadership affects all emotional outcomes uniformly and suggest that intervention strategies should be tailored to specific leadership dimensions and symptom targets. Below, we discuss the theoretical implications of these distinct pathways, compare our findings with existing literature, and address the practical significance and limitations of this study.

### Humiliating behavior as a critical toxic leadership dimension

Among the four dimensions of toxic leadership, humiliating behavior demonstrated the strongest correlation with nurses' stress responses (edge weight = 0.27). This finding is consistent with previous research indicating that dignity-threatening behaviors are particularly detrimental to employee wellbeing (59, 60). From the perspective of social exchange theory, humiliating behavior disrupts the fundamental balance of workplace social exchange, leaving nurses in a state of threatened psychological safety that triggers chronic stress reactions (61). This effect may be amplified in healthcare settings, where high-intensity work environments heighten nurses' sensitivity to negative managerial behaviors (62). Unlike previous studies that treated toxic leadership as a unidimensional construct (57, 58), our findings highlight the unique contribution of humiliating behavior, suggesting that interventions should specifically target this dimension. However, it is worth noting that while social exchange theory provides a useful framework, it does not explain why some nurses appear resilient to humiliating behaviors while others are highly susceptible. Individual differences such as coping styles, prior trauma history, or organizational tenure may moderate this relationship (63), yet these factors were not examined in the current study. Future longitudinal research should test whether reductions in humiliating behavior precede decreases in stress symptoms, and explore potential moderators of this relationship.

### Intemperate behavior and depressive symptoms

A significant association was also identified between intemperate behavior and depressive symptoms (edge weight = 0.15). According to Tepper's theoretical framework of destructive leadership (64), intemperate leadership operates through dual mechanisms: emotional contagion, whereby managers' negative emotions are directly transmitted to subordinates (65), and cognitive appraisal, whereby nurses interpret such behavior as a lack of organizational support, leading to feelings of helplessness and reduced self-efficacy (66). These findings extend previous work by specifying that not all toxic behaviors equally affect all emotional outcomes; intemperate behavior appears particularly relevant to depression, whereas humiliating behavior is more strongly linked to stress. However, several points regarding this finding warrant attention. First, the dual-mechanism explanation (emotional contagion and cognitive appraisal) assumes that these processes operate independently, yet they may interact or reinforce each other in ways that our cross-sectional data cannot disentangle. For example, negative affect transmitted through emotional contagion might amplify cognitive appraisals of organizational neglect, creating a synergistic effect that our edge weight (0.15) may underestimate. Second, the framework does not account for protective factors such as peer support, supervisory backup, or organizational justice, which may buffer the impact of intemperate behavior on depression (67). The relatively modest edge weight (0.15) suggests that substantial variance remains unexplained, and future research should examine whether these buffering factors moderate the relationship between intemperate behavior and depression. Longitudinal studies with repeated measurements are needed to test whether changes in intemperate behavior temporally precede changes in depressive symptoms, and to explore potential bidirectional effects.

### Anxiety as a central hub in the emotional symptom network

The results revealed that anxiety exhibited the highest node strength, forming strong connections with both depression (edge weight = 0.34) and stress (edge weight = 0.34). This central hub position suggests that anxiety may serve as a core driver of emotional distress among nurses, influencing overall mental health through multiple pathways. This finding aligns with the theory of emotional contagion (68), which posits that anxiety diffuses within healthcare teams by increasing cognitive load and diminishing self-efficacy (69). Compared with previous studies that examined anxiety in isolation (70, 71), our network approach reveals its interconnectedness with other emotional symptoms, underscoring the importance of targeting anxiety to achieve broader improvements in mental health. However, while emotional contagion theory emphasizes the transmission of affect from leaders to followers, our cross-sectional data cannot distinguish between contagion effects and shared environmental influences. Future research should adopt longitudinal study designs. By repeatedly measuring nurses' anxiety levels and organizational environmental variables at multiple time points, researchers can examine whether the spread of anxiety remains significant after controlling for shared environmental factors, thereby providing a more accurate estimate of the true effect size of interpersonal emotional contagion.

### Stress as a key bridge node

Notably, stress exhibited unique network characteristics: while its node strength was relatively low (strength = 0.42), it demonstrated the highest betweenness centrality (betweenness = 0.89), indicating its critical “bridging” role between toxic leadership behaviors and emotional symptoms. This finding is highly consistent with Hobfoll's Conservation of Resources Theory (72, 73), which proposes that persistent stress leads to the gradual depletion of psychological resources, triggering cascading effects such as depression and occupational burnout (74–76). The bridging role of stress suggests that interventions targeting stress reduction may have ripple effects across the entire symptom network, potentially alleviating both anxiety and depression. However, Conservation of Resources Theory emphasizes the objective loss of resources. Such as time, autonomy, and social support, yet our study did not directly measure resource depletion. We inferred resource loss from stress symptoms, which is an indirect proxy at best. Future research should incorporate direct measures of resource loss, for example perceived organizational support scales, work autonomy measures, or objective workload indicators, to test whether resource depletion statistically mediates the relationship between toxic leadership and stress.

### Comparison with existing literature

The present findings both corroborate and extend existing research. Consistent with studies by Abdelrahman et al. (77) and Labrague et al. (78), we confirm the presence of toxic leadership behaviors in healthcare settings and their negative impact on nurses' mental health. However, our network approach reveals nuances that traditional variable-centered methods cannot capture. For instance, while previous research has broadly associated toxic leadership with increased burnout and turnover intentions (79, 80), our findings specify that humiliating behavior is most strongly linked to stress, while intemperate behavior is more closely associated with depression. This granularity has important implications for intervention design.

### Theoretical and practical implications

Theoretically, this study contributes to the literature by demonstrating the utility of network analysis for understanding complex relationships between leadership behaviors and employee mental health. The identification of specific pathways between toxic leadership dimensions and emotional symptoms extends social exchange theory, conservation of resources theory, and the theory of emotional contagion by specifying the precise mechanisms through which leadership affects mental health.

From a practical standpoint, the findings offer actionable guidance for healthcare organizations. Rather than implementing broad, untargeted interventions, hospitals may focus on reducing specific toxic behaviors, particularly humiliating behavior, through manager training programs that emphasize respectful communication and emotional regulation. Concurrently, stress management interventions for nurses, such as cognitive-behavioral therapy-based programs and routine mental health monitoring, may serve as effective entry points for disrupting the negative cycle between leadership behaviors and emotional distress. Given the central role of anxiety in the symptom network, anxiety reduction programs may also yield cascading benefits for depression and stress.

## Conclusion

This study employed network analysis to elucidate the complex structural relationships between nursing managers' toxic leadership behaviors and nurses' anxiety, depression, and stress. The findings reveal that humiliating behavior represents the most influential toxic leadership dimension, while stress serves as the key bridge connecting leadership behaviors to emotional symptoms. These insights extend existing theoretical frameworks by specifying the precise pathways through which negative leadership affects nurses' psychological wellbeing. From a practical standpoint, the results offer focused guidance for healthcare organizations: interventions targeting the reduction of humiliating behaviors among nursing managers, combined with stress management programs for nurses, may provide the most efficient approach to disrupting the negative cycle between leadership practices and emotional distress.

### Limitations

First, the cross-sectional design restricts the ability to establish causal relationships between toxic leadership behaviors and nurses' anxiety, depression, and stress; future research should employ longitudinal tracking designs combined with network analysis methods for deeper investigation. Second, the sample was drawn exclusively from two cities (Zunyi and Guiyang) in southwestern China, which may limit the generalizability of findings to other regions or healthcare systems, future studies should employ multicenter designs with larger and more diverse samples to enhance generalizability. Third, while we collected demographic information, we did not account for all potential confounding factors such as work hours, shift schedules, or family responsibilities, which may influence nurses' emotional states. Fourth, the inclusion criterion of at least 1 year of nursing experience, while intended to ensure stable perceptions of leadership behaviors, may limit the applicability of findings to newly hired nurses. Fifth, due to the study design, this paper did not conduct separate analyses for specific departments (such as ICU or emergency room nurses), future research plans include comparative analyses across different groups. Finally, the proposed intervention targets identified in this study have not been empirically tested; subsequent intervention studies are needed to verify the effectiveness of these targets before they can be applied in clinical practice. Additionally, due to the cross-sectional design, network centrality indices (e.g., bridge expected influence) should be interpreted as statistical associations rather than causal pathways. The identified bridging nodes are intended to generate hypotheses for future longitudinal or intervention studies, and should not be construed as validated intervention targets based on the current data.

## Data Availability

The original contributions presented in the study are included in the article/Supplementary material, further inquiries can be directed to the corresponding author.
